# Oxygen administration for patients with ARDS

**DOI:** 10.1186/s40560-021-00532-0

**Published:** 2021-02-06

**Authors:** Shinichiro Ohshimo

**Affiliations:** Department of Emergency and Critical Care Medicine, Graduate School of Biomedical and Health Sciences, Hiroshima University, 1-2-3 Kasumi, Minami-ku, Hiroshima, 734-8551 Japan

**Keywords:** Acute respiratory failure, Mechanical ventilation, Extracorporeal membrane oxygenation, High-flow nasal cannula, Non-invasive positive pressure ventilation, Prognosis, Complication

## Abstract

Acute respiratory distress syndrome (ARDS) is a fatal condition with insufficiently clarified etiology. Supportive care for severe hypoxemia remains the mainstay of essential interventions for ARDS. In recent years, adequate ventilation to prevent ventilator-induced lung injury (VILI) and patient self-inflicted lung injury (P-SILI) as well as lung-protective mechanical ventilation has an increasing attention in ARDS.

Ventilation-perfusion mismatch may augment severe hypoxemia and inspiratory drive and consequently induce P-SILI. Respiratory drive and effort must also be carefully monitored to prevent P-SILI. Airway occlusion pressure (*P*_0.1_) and airway pressure deflection during an end-expiratory airway occlusion (*P*_occ_) could be easy indicators to evaluate the respiratory drive and effort. Patient-ventilator dyssynchrony is a time mismatching between patient’s effort and ventilator drive. Although it is frequently unrecognized, dyssynchrony can be associated with poor clinical outcomes. Dyssynchrony includes trigger asynchrony, cycling asynchrony, and flow delivery mismatch. Ventilator-induced diaphragm dysfunction (VIDD) is a form of iatrogenic injury from inadequate use of mechanical ventilation. Excessive spontaneous breathing can lead to P-SILI, while excessive rest can lead to VIDD. Optimal balance between these two manifestations is probably associated with the etiology and severity of the underlying pulmonary disease.

High-flow nasal cannula (HFNC) and non-invasive positive pressure ventilation (NPPV) are non-invasive techniques for supporting hypoxemia. While they are beneficial as respiratory supports in mild ARDS, there can be a risk of delaying needed intubation. Mechanical ventilation and ECMO are applied for more severe ARDS. However, as with HFNC/NPPV, inappropriate assessment of breathing workload potentially has a risk of delaying the timing of shifting from ventilator to ECMO. Various methods of oxygen administration in ARDS are important. However, it is also important to evaluate whether they adequately reduce the breathing workload and help to improve ARDS.

## Introduction

Acute respiratory distress syndrome (ARDS) is a fatal condition with insufficiently clarified etiology. The Berlin Definition is the currently available clinical criteria for ARDS [1], which consists of acute onset, hypoxia with the partial pressure of arterial oxygen (PaO_2_)/fraction of inspiratory oxygen (F_I_O_2_) ratio of less than 300 mmHg, diffuse infiltrates on chest radiograph or computed tomography (CT), and respiratory failure not fully explained by the cardiac failure or fluid overload. The survival rate is improving from the first report of 50% [2] to around 70% [3, 4]. However, the precise survival rate remains uncertain, because the pathogenesis of ARDS is very heterogeneous and the survival rate may vary according to the pathogenesis of ARDS.

Supportive care for severe hypoxemia remains the mainstay of an effective intervention for ARDS. An adequate oxygen administration to prevent hypoxemia, as well as ventilator-induced lung injury (VILI) and patient self-inflicted lung injury (P-SILI), could potentially facilitate recovery of alveolar epithelial damage in ARDS.

In this review, I focused on the recent advances in the field of oxygen administration for patients with ARDS.

## Unclarified issues

The Berlin definition is the most widely used severity classification for ARDS [5]. According to this definition, potential therapeutic options according to the severity of ARDS have been proposed [6]. In this proposal, it is stated that ARDS requires low tidal volume ventilation of 6 mL/kg predicted body weight and plateau pressure below 30 cm H_2_O regardless of severity. For mild to moderate ARDS, low to moderate positive end-expiratory pressure (PEEP) is suggested, and for more severe ARDS, higher PEEP is needed. Non-invasive ventilation is suggested for mild ARDS with a PaO_2_/F_I_O_2_ ratio of 200 or higher. In more severe ARDS with a PaO_2_/F_I_O_2_ ratio of less than 150, inhibition of excessive spontaneous breathing with muscle relaxants and supine positioning to improve ventilation-perfusion mismatch are recommended. These recommendations are very reasonable and acceptable. However, these recommendations are based only on the severity of ARDS (i.e., PaO_2_/F_I_O_2_ ratio) and do not take into account other factors, for example, P-SILI due to excessive spontaneous breathing, reduction of respiratory workload, patient-ventilator dyssynchrony that may potentially cause alveolar epithelial injury, and, conversely, ventilator-induced diaphragm dysfunction (VIDD) complicated by ventilator over/under-assistance, dyssynchrony, or excessive PEEP. For achieving adequate respiratory support in ARDS, factors other than the PaO_2_/F_I_O_2_ ratio may need to be considered.

**Fig. 1:**
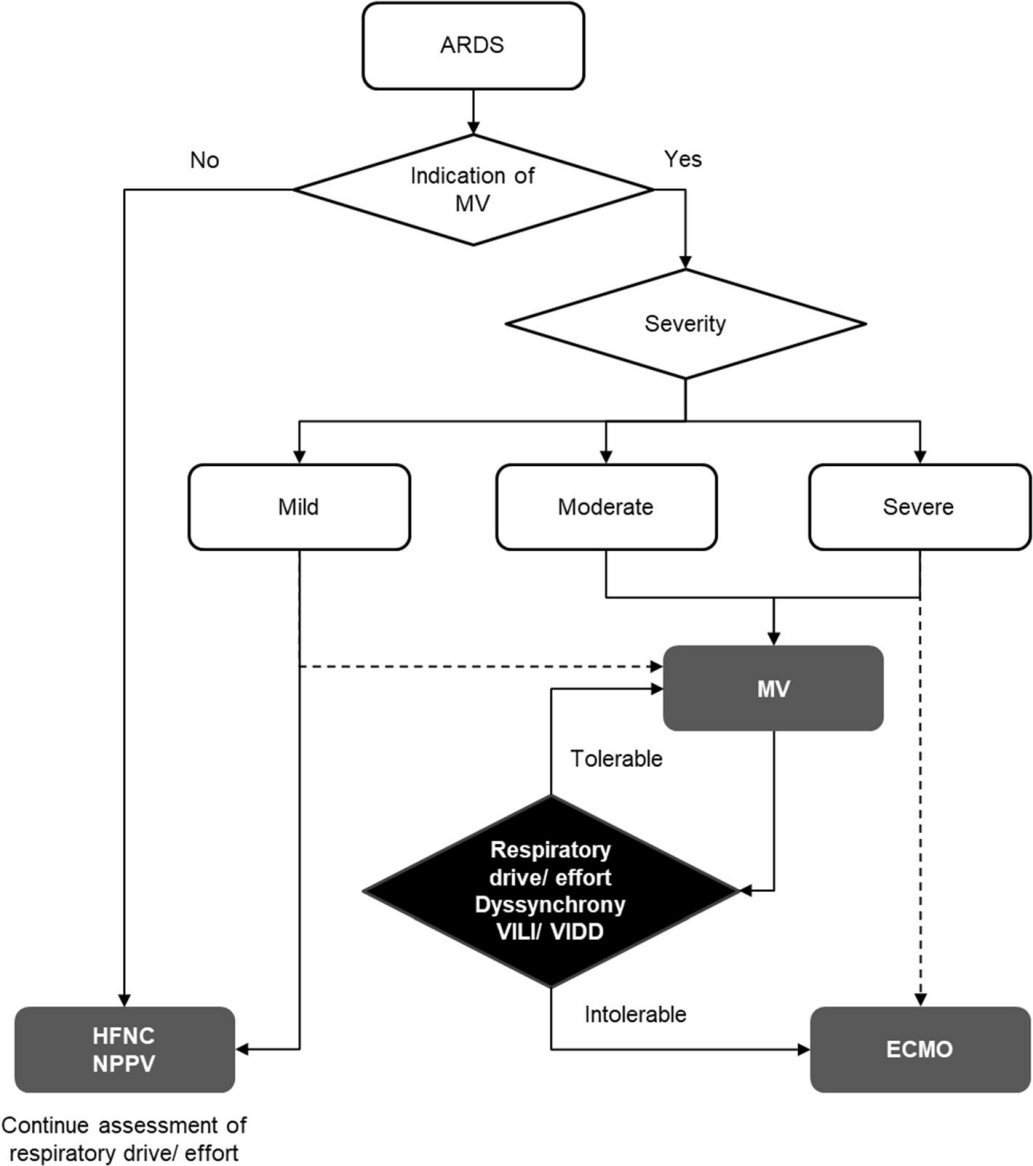
Management flow of oxygen administration for patients with ARDS. According to the severity of ARDS, HFNC, NPPV, MV, and ECMO should be used as appropriate. The most important thing is to continuously monitor not only oxygenation, but also respiratory drive/ effort and other parameters. If any of these indicators are inadequate, the procedure of oxygen administration should be immediately changed for improving respiratory drive/ effort. ARDS, acute respiratory distress syndrome; HFNC, high-flow nasal cannula; NPPV, non-invasive positive pressure ventilation; MV, mechanical ventilation; ECMO, extracorporeal membrane oxygenation

Figure 1 summarizes the suggested flowchart of respiratory management in patients with ARDS. Various physiological variables including respiratory drive and effort, dyssynchrony, and VIDD should be considered for the adequate management of hypoxemia in ARDS.

## Respiratory drive and effort

Excessive respiratory drive and effort may increase the severity of lung injury, which may prolong the duration of mechanical ventilation and affect patient outcomes. Therefore, adequate regulation of spontaneous breathing has been increasingly recognized as important in respiratory management in ARDS. It would be important to systematically measure the respiratory drive in ventilated patients making spontaneous breathing and to assess how high respiratory drive leads to subsequent deterioration of respiratory function during attempts to weaning from mechanical ventilation [7].

**Fig. 2:**
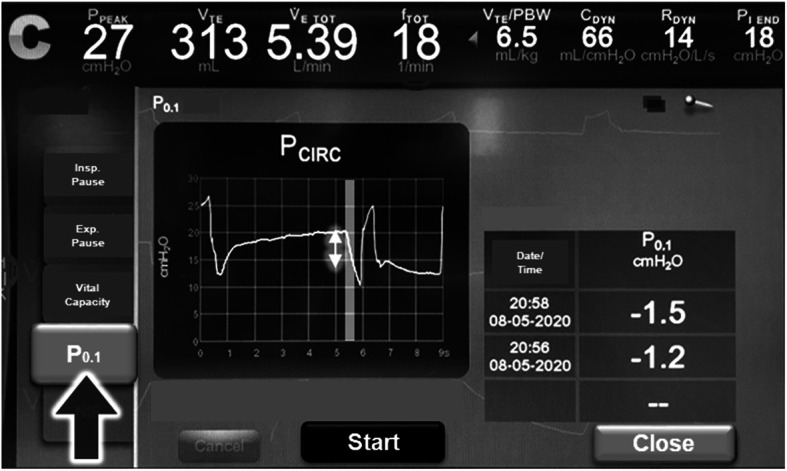
Procedure to measure *P*_0.1_. Ventilator screen displaying *P*_0.1_ calculated from the change in airway pressure associated with 0.1 s of airway occlusion. Gray arrow indicates the buttons used for *P*_0.1_ measurement. Gray square indicates the intervals of 0.1 s of airway occlusion. White arrow indicates the negative airway pressure occurring during 0.1 s of airway occlusion (*P*_0.1_), with the most recent data being − 1.5 cm H_2_O

Airway occlusion pressure (*P*_0.1_) is a simple, non-invasive measure for estimating respiratory drive during mechanical ventilation, which can be used automatically in almost all mechanical ventilators [8, 9]. *P*_0.1_ is defined as the negative airway pressure occurring during the first 0.1 s of an occluded inspiration (Fig. 2). The absence of airflow rate makes *P*_0.1_ independent from respiratory compliance, resistance, and muscle weakness. Small *P*_0.1_ indicates the weak patient’s inspiratory effort, while large *P*_0.1_ indicates the strong patient’s inspiratory effort. Too weak inspiratory effort can be associated with weaning failure, while too strong inspiratory effort can be associated with P-SILI. Telias et al. [10] investigated the correlation of *P*_0.1_ with respiratory drive and effort by using various mechanical ventilators. *P*_0.1_ was well correlated with alternative measures of respiratory drive, including the electrical activity of the diaphragm, and with inspiratory effort measured by the esophageal pressure-time product. In addition, *P*_0.1_ could have detected excessive inspiratory effort with the cut-off value of 3.5 to 4.0 cm H_2_O (sensitivity, 0.67; specificity, 0.86–0.91; accuracy 0.82–0.86) and low inspiratory effort with the cut-off value of 1.0 cm H_2_O (sensitivity, 0.75; specificity, 0.95; accuracy 0.89), respectively. It is important to note that Servo ventilators estimate *P*_0.1_ by the airway pressure drop during the trigger phase without true occlusions, whereas others (Evita-XL and Puritan Bennett) measure *P*_0.1_ with true occlusions, suggesting that *P*_0.1_ can be underestimated in the use of Servo ventilators. In that case, the cut-off value for *P*_0.1_ should be lower (i.e., 2.0 cm H_2_O).

## Dyssynchrony

**Fig. 3:**
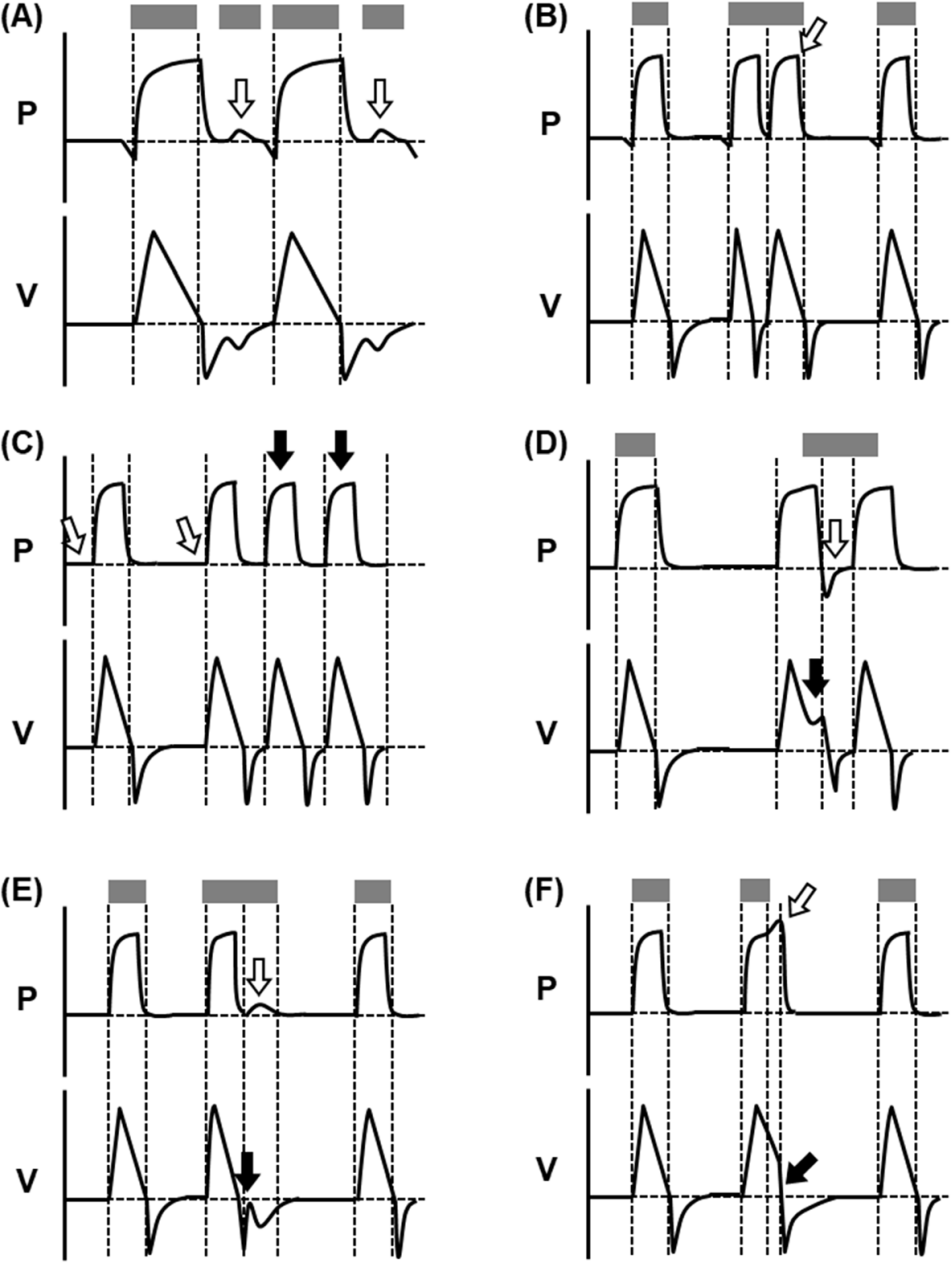
Variation of dyssynchrony. Combination of airway pressure and tidal volume curves showing various types of dyssynchrony. **a** Ineffective triggering (miss triggering): Small waveform changes during expiration indicate the presence of weak spontaneous breathings that was not triggered (white arrows). It can be associated with a weak patient respiratory drive. Ineffective triggering can occur in situations combined with underlying auto-PEEP and excessive ventilation. It can be adjusted by extended expiratory time and increased trigger sensitivity. **b** Double triggering: Prolonged spontaneous breathing is generating a second mechanical ventilation immediately after the completion of the first mechanical ventilation (white arrow). It can occur when second breathing starts before the first ventilation delivery has completed. Double triggering may be adjusted by extended inspiratory time. **c** Auto triggering: Excessive mechanical ventilation (black arrows) is occurring despite the lack of patient’s respiratory effort (white arrows), which might be associated with the water in the circuit or circuit leakage. This type of asynchrony can occur in cases of sputum in the circuit, condensation, circuit leakage, or heart oscillations. Auto triggering can be adjusted by lowering the triggering sensitivity of the mechanical ventilator or by removing sputum or condensation. **d** Reverse triggering: Spontaneous breathing is paradoxically triggered by mechanical ventilation, resulting in a generation of double triggering (white and black arrows). Reverse triggering is frequently observed in highly sedated patients. Reverse triggering can be adjusted by decreasing sedation. **e** Premature cycling: Since mechanical ventilation is completed earlier than the completion of spontaneous breathing, there remains spontaneous breathing in the expiratory phase. Waveform swinging is observed in the exhalation phase (white and black arrows). Premature cycling can be adjusted by extending inspiratory time. **f** Delayed cycling: Since spontaneous breathing is completed earlier than the completion of mechanical ventilation, the inspiratory phase is rapidly terminated (white and black arrows). Delayed cycling can be adjusted by reducing inspiratory time. Gray bars indicate the time periods of spontaneous breathing. P, airway pressure curve; V, tidal volume curve

Patient-ventilator dyssynchrony is a time mismatching between patient’s effort and ventilator drive, often occurs during assisted ventilator modes. Although dyssynchrony can occur in around 25% of mechanically ventilated patients [11], it is frequently unrecognized and is associated with poor clinical outcomes. Esophageal pressure (transpulmonary pressure) can be used for better detection of dyssynchrony. Dyssynchrony can lead to increased work of breathing, auto-positive end-expiratory pressure (PEEP), poor gas exchange, prolonged use of mechanical ventilation, and barotrauma [11, 12]. The major groups of dyssynchrony include (A) trigger asynchrony, (B) cycling asynchrony, and (C) flow delivery mismatch [13]. Adjusting the ventilator settings can dramatically improve patient-ventilator dyssynchrony [14] (Fig. 3).

**Fig. 4:**
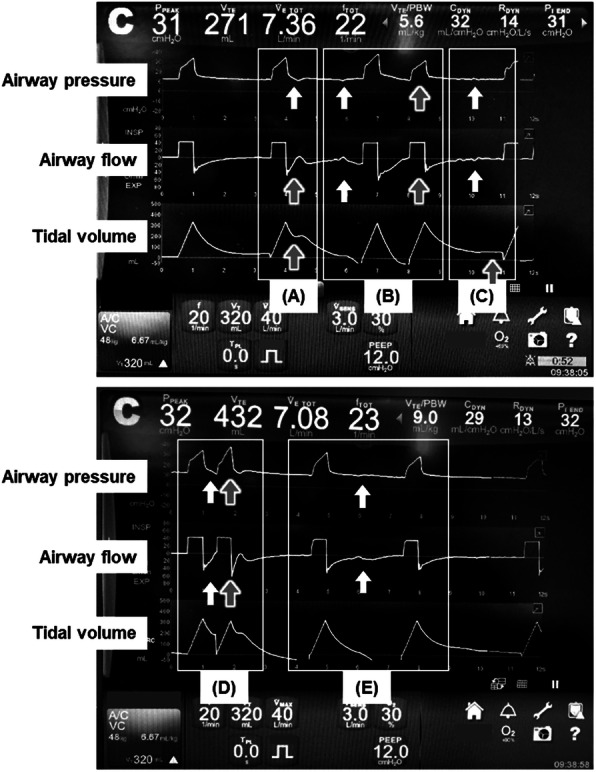
Practical examples of various dyssynchrony. Ventilator screen displaying various dyssynchrony. **a** Reverse triggering: The airway pressure curve demonstrated the waveform during the expiratory phase swinging slightly toward negative pressure (white arrows). The waveforms of airway flow and tidal volume were also deformed (gray arrows), indicating a presence of reverse triggering. **b** Ineffective triggering (white arrow) and auto triggering (gray arrow): The baseline before the first inspiration fluctuated slightly (white arrows), indicating a presence of ineffective triggering. The second inspiration had a different waveform from the others (gray arrows) with the absence of the patient’s respiratory effort, indicating auto triggering. **c** The airway pressure/airway flow curve presented a jaggy baseline (white arrows), indicating a presence of droplets or secretion in the circuit. The ventilation volume curve did not return to the baseline (gray arrow), indicating a presence of auto-PEEP. **d** The first inspiration induced reverse triggering and deformed expiratory waveform (white arrow). Consequently, a reverse triggering induced a double triggering (gray arrow). **e** The airway pressure/airway flow curve demonstrated the first expiratory waveform was trembling (white arrow), indicating the presence of an ineffective triggering

Figure 4 shows a patient presenting with various dyssynchronies. The patient exhibited a great variety of dyssynchronies, including ineffective triggering, reverse triggering, double triggering, and auto triggering in a very short period of time. The target tidal volume was set as 320 mL (6 mL/kg), and the actual tidal volume was 271 mL (5.6 mL/kg). The observed dyssynchronies increased the tidal volume up to 432 mL (9.0 mL/kg), which was unacceptable for lung-protective ventilation.

Among these dyssynchronies, reverse triggering is the newly emerging type of dyssynchrony with a potential risk of alveolar injury. After the widespread use of low tidal volume ventilation in ARDS, double triggering in heavily sedated patients can be frequently observed in association with breath stacking [15]. Greater neural inspiratory time compared with ventilatory inflation time could be responsible for this phenomenon. Recent studies suggested that one-third of the breath stacking was associated with reverse triggering [16]. Reverse triggering is likely to be injurious if the ventilator delivers a second breath. Greater effort resulting from reverse triggering can be injurious as it can induce eccentric contractions when it occurs during the expiratory phase and can induce pendelluft and excessive local lung stress when it occurs during the inspiratory phase [17–19].

On the contrary, Rodriguez et al. [20] reported inconsistent findings in a prospective, multicenter, observational study. They investigated the frequency of reverse triggering and its impact on clinical outcomes during the early phase of non-paralyzed mechanically ventilated patients with ARDS. They collected a total of 100 patients and evaluated the ventilatory patterns obtained after a median of 24 h of intubation. Fifty percent of patients demonstrated reverse triggering without breath stacking. Increased occurrence of reverse triggering was associated with lower tidal volume (odds ratio [OR], 0.91 per 0.1 mL/kg; 95% confidence interval (CI), 0.84–0.98; *p* = 0.02) and less use of fentanyl (OR, 0.93 per 10 micrograms; 95%CI, 0.88–0.99; *p* = 0.02). We should note that the presence of reverse triggering was not associated with the duration of mechanical ventilation, but was associated with a reduced 90-day in-hospital mortality rate (hazard ratio [HR], 0.65; 95%CI, 0.57–0.73; *p* < 0.001). The authors concluded that the early detection of reverse triggering may be a good indicator of a favorable outcome in patients with mild to moderate ARDS. Further investigations are necessary for determining whether specific intervention should be applied for reverse triggering to improve patient outcomes.

## Ventilator-induced diaphragm dysfunction (VIDD)

Ventilator-induced diaphragm dysfunction (VIDD) is a form of iatrogenic injury from inadequate use of mechanical ventilation [21, 22]. VIDD is not solely due to modes of mechanical ventilation but rather can be caused by inappropriate ventilator support through various mechanisms. While VIDD can be largely induced by disuse atrophy due to ventilator over-assistance, several other mechanisms including load-induced injury due to ventilator under-assistance, eccentric contractile injury due to dyssynchrony, and excessive shortening due to high PEEP can also be involved.

Diaphragm muscle weakness can rapidly occur in mechanically ventilated critically ill patients in the ICU [23–26], which can be associated with an increased mortality rate [27–29]. VIDD can occur twice as frequently as limb muscle weakness, which has a negative impact on successful weaning [30]. Disuse of diaphragm muscle can be a potential risk factor of VIDD. However, the best balance between the increased respiratory drive/ effort and the risk of VIDD can be difficult to be adequately defined. Diaphragm ultrasound can directly visualize diaphragm, which has been proposed to assess VIDD and a resultant inspiratory effort [26, 31–33]. Low excursion of the diaphragm or weak apposition thickening detected by diaphragm ultrasound was a strong predictor of weaning failure [30, 33–36].

The impact of spontaneous mechanical ventilation on VIDD has not been well known. Animal studies have demonstrated that spontaneous mechanical ventilation may be more protective than controlled mechanical ventilation [37, 38], whereas these data have not been well confirmed in human subjects. Marin-Corral et al. [39] investigated the effects of different modes of mechanical ventilation in respiratory and peripheral muscles in ventilated organ donors. They demonstrated that the cross-sectional area of the diaphragm was significantly reduced in patients who did not receive diaphragm stimuli compared with those who received diaphragm stimuli. These findings supported the hypothesis that spontaneous mechanical ventilation would be more favorable for preventing VIDD compared with controlled mechanical ventilation.

Lindqvist et al. [40] investigated the effect of PEEP on VIDD. Typically, mechanically ventilated patients with ARDS would be managed by using PEEP to avoid alveolar collapse. However, the use of PEEP can increase end-expiratory lung volume, resulting in the flattened diaphragm dome and structural modification in the diaphragm fibers. They demonstrated that PEEP induced a caudal movement of the diaphragm both in critically ill patients and in animal models, which resulted in the shortening of muscle fiber length. The shortened muscle fibers can generate less force, resulting in diaphragm dysfunction. Slow reduction in PEEP would be favorable to allow the reversal of longitudinal muscle atrophy.

Goligher et al. [41] investigated the clinical impact of VIDD on the duration of mechanical ventilation. They included a total of 191 patients requiring mechanical ventilation, and diaphragm thickness was measured daily by ultrasound. More than a 10% decrease in diaphragm thickness was observed in 41% of patients by median day 4. Development of decreased diaphragm thickness was associated with a lower probability of ventilator withdrawal (HR, 0.69 per 10% reduction; 95% CI, 0.54–0.87), prolonged ICU stays (adjusted duration ratio, 1.71; 95%CI, 1.29–2.27), and increased complications (OR, 3.00; 95%CI, 1.34–6.72). On the other hand, however, the development of increased diaphragm thickness was also associated with prolonged ventilation (adjusted duration ratio, 1.38; 95%CI, 1.00–1.90). Patients with a diaphragm thickening rate of 15 to 30% (similar to resting breathing) during the first 3 days presented the shortest duration of ventilation. This study suggested that targeting an inspiratory effort level similar to that of healthy subjects at rest might be most effective for reducing the duration of mechanical ventilation.

## HFNC and NPPV

High-flow nasal cannula (HFNC) and non-invasive positive pressure ventilation (NPPV) are the non-invasive techniques for supporting hypoxemia and respiratory effort in patients with severe hypoxemic respiratory failure. The major advantages of HFNC could be the better tolerability compared with NPPV, provides high F_I_O_2_, and can unload the inspiratory muscles by washing out the dead space in the upper airway with low levels of positive pressure. On the other hand, the major advantages of NPPV could be the provision of higher F_I_O_2_, and more reliable unloading of the inspiratory muscles by providing higher positive airway pressure. HFNC and NPPV can be effective non-invasive ventilatory devices for ARDS, since the reduction of respiratory workload is an important factor as well as the improvement of hypoxemia [42, 43].

As a result of the increasing use of HFNC, there is a risk of delays in needed intubation. This is an important concern, as a number of previous studies demonstrated that patients who failed to manage NPPV with acute respiratory failure have worse outcomes. This has been shown in NPPV [29] and also in HFNC [44]. In addition, there are no prospectively validated criteria for intubation in patients with ARDS. This may result in considerable variation among clinicians and may affect patient outcomes.

Roca et al. [45] investigated the diagnostic accuracy of the ROX index defined as the ratio of oxygen saturation (SpO_2_)/F_I_O_2_ to respiratory rate for predicting the need for intubation in patients with hypoxemic respiratory failure. They included a total of 191 patients, of whom 68 (36%) patients required intubation. The area under the ROC curve of ROX index at 2, 6, and 12 h after initiating HFNC for predicting intubation were 0.648 (95%CI, 0.561–0.734; *p* = 0.001), 0.672 (95%CI, 0.580–0.764; *p* < 0.001), and 0.695 (95%CI, 0.598–0.791; *p* < 0.001), respectively. A ROX index of < 2.85 at 2 h, < 3.47 at 6 h, and < 3.85 at 12 h of initiating HFNC, respectively, were predictors of HFNC failure. In addition, the serial changes of its value may help discriminate patients with HFNC success from those with HFNC failure.

Another important topic regarding HFNC is the identification of subgroups of acute respiratory failure for which HFNC is effective. Azoulay et al. [46] investigated whether HFNC decreases mortality among immunocompromised patients with ARDS compared with standard oxygen therapy. They included a total of 776 patients with a median partial pressure of arterial oxygen (PaO_2_)/F_I_O_2_ ratio of 136 (interquartile range [IQR], 96–187), and 128 (IQR, 92–164) in the intervention and control groups, respectively. The authors found no significant difference between the cohorts regarding 28-day mortality (36% vs 36%), intubation rate (39% vs 44%), ICU length of stay (8 vs 6 days), ICU-acquired infections (10% vs 11%), or hospital length of stay (24 vs 27 days). These results suggested that intensive attention for improving oxygenation may not beneficial for improving survival in immunocompromised patients with ARDS.

The HACOR (heart rate, acidosis, consciousness, oxygenation, and respiratory rate) score was an index for predicting NPPV failure in patients with ARDS. Duan et al. [47] included a total of 449 patients requiring NPPV and evaluated the predictive value of the HARCOR score. The failure rate of NPPV was 48% and 39% in the test and validation cohorts, respectively. The areas under the ROC curve of the HACOR score assessed at 1 h of NPPV for predicting HPPV failure were 0.88 (95%CI, 0.84–0.91) for the test cohort and 0.91 (95%CI, 0.88–0.94) for the validation cohort. With the cut-off value of > 5, sensitivity, specificity, and diagnostic accuracy were 73–76%, 90–93%, and 82–86%, respectively. In addition, the serial changes of its value could also discriminate patients with NPPV success from those with NPPV failure.

Innocenti et al. [48] further investigated the utility of the HACOR score in a patient with acute respiratory failure requiring NPPV. They retrospectively included a total of 644 hypoxemic patients with or without hypercapnia, of whom 147 (23%) patients have died during the observation period. The area under the ROC curve of the HACOR score for predicting in-hospital mortality were 0.64 (95%CI, 0.58–0.69; *p* < 0.001) at initiating NPPV, 0.68 (95%CI, 0.63–0.73; *p* < 0.001) at 1 h of NPPV, and 0.75 (95%CI, 0.70–0.80; *p* < 0.001) at 24 h of NPPV, respectively. The Cox survival analysis for in-hospital mortality demonstrated that the HACOR score of > 5 at 24 h of NPPV was associated with increased in-hospital mortality (RR, 2.39; 95%CI 1.60–3.56; *p* < 0.001).

Since HFNC or NPPV can be used outside the ICU and could be a good alternative for patients who are not candidates for invasive mechanical ventilation, these scoring tools would be useful for the adequate use of HFNC/ NPPV.

## Mechanical ventilation

Because mechanical ventilation includes a potential risk of VILI, low tidal volume ventilation, synonymous with lung-protective ventilation, remains the most recommended ventilatory management in patients with ARDS. Previous studies and meta-analyses demonstrated the beneficial effect of low tidal volume ventilation in patients with ARDS. This ventilatory strategy typically consists of tidal volume of 4–8 mL/kg predicted body weight, plateau pressure (*P*_plat_) of < 30 cm H_2_O, and sufficient PEEP, which can be roughly estimated as multiplying F_I_O_2_ by 20 (i.e., if F_I_O_2_ is 0.5, PEEP would be around 10 cm H_2_O; if F_I_O_2_ is 1.0, PEEP would be around 20 cm H_2_O), according to the PEEP/F_I_O_2_ table [49]. A randomized clinical trial (RCT) including 961 patients with ARDS, the PReVENT trial [50], investigated the benefit of low tidal volume ventilation (< 6 mL/kg of predicted body weight (PBW), with titration to a target of 4 mL/kg PBW) compared with intermediate tidal volume ventilation (10 mL/kg PBW). They demonstrated no significant benefit of low tidal volume ventilation in ventilator-free days, hospital length of stay, 28-day and 90-day mortalities, or the risk of adverse events. However, around 25% of the low tidal volume ventilation cohort received a tidal volume of > 6 mL/kg PBW by day 3. Similarly, around 25% of the intermediate tidal volume ventilation cohort received a tidal volume of > 10 mL/kg PBW by day 3. Therefore, insufficient differences observed in this study could have been associated with these actual tidal volumes.

The LIVE study [51] was a multicenter, single-blind, stratified, parallel-group, RCT enrolling patients with moderate to severe ARDS in France. They investigated whether a mechanical ventilation strategy that was personalized to individual patients’ lung morphology would improve survival when compared with standard care. In the personalized cohort, patients with focal ARDS received a tidal volume of 8 mL/kg, low PEEP, and prone position. Patients with non-focal ARDS received a tidal volume of 6 mL/kg, along with recruitment maneuvers and high PEEP. In a multivariate analysis, there was no difference in 90-day mortality between the cohorts (HR, 1.01; 95%CI, 0.61–1.66; *p* = 0.98). However, a significant number of misclassifications of focal or non-focal ARDS was found in 21% of enrolled patients. In addition, misclassification of patients was associated with the increased 90-day mortality compared with the control cohort (HR, 2.8; 95%CI, 1.5–5.1; *p* = 0·012). This study suggested the potential difficulty and risk of personalized ventilatory management.

The difference between volume-limited assist-control mode and pressure-limited assist-control mode seems minimal. Previous studies demonstrated no significant differences in mortality, oxygenation, or respiratory effort [52–54]. Synchronized intermittent mandatory ventilation (SIMV) is one of the most frequently used ventilatory modes to facilitate patient-ventilator synchrony, to preserve respiratory muscle, and to minimize the risk of auto-PEEP. However, in terms of respiratory effort and constant tidal volume, the assist-control mode would be more reasonable for patients with ARDS compared with SIMV. Pressure support ventilation (PSV) could be an adequate option for weaning from mechanical ventilation, because respiratory effort can be proportionally supported by PSV. However, no studies supported the beneficial effect of PSV for weaning to date. In addition, the following characteristics of PSV could be disadvantages for patients with the acute phase of ARDS:

1. Respiratory effort of patients with ARDS cannot be fully supported.
2. Defined tidal volume and respiratory rate cannot be provided.
3. Patient-ventilator asynchrony can occur.
4. Higher levels of pressure support could be necessary to prevent alveolar collapse.

Regardless of whether the volume-limited or pressure-limited assist-control mode is selected, fully supported modes are generally favorable compared with partially supported modes in patients with ARDS.

## Oxygen concentration and oxygen toxicity

Most patients with ARDS require a high concentration of oxygen to maintain sufficient oxygenation. However, we should be aware of the potential toxicity of a high concentration of oxygen. Previous studies using human cohort and animals demonstrated that the high concentration of inspired oxygen was associated with the occurrence of acute lung injury, ranging from mild to severe diffuse alveolar damage (DAD) [55]. In general, oxygen toxicity may be increased in patients receiving F_I_O_2_ of more than 0.6. A longer duration of exposure can induce severer lung injury.

Chu et al. [56] conducted a meta-analysis to investigate the impacts of liberal and conservative oxygen therapy on mortality and morbidity in acutely ill adult patients. They included a total of 25 RCTs with 16,037 patients and demonstrated that the liberal oxygen strategy (median F_I_O_2_, 0.52, IQR 0.28–1.0) increased in-hospital mortality and 30-day mortality compared with the conservative oxygen strategy (median F_I_O_2_, 0.21; IQR, 0.21–0.25). In addition, a meta-regression effect of SpO_2_ on in-hospital mortality showed an increased SpO_2_ was associated with an increased risk of mortality. Barbateskovic et al. [57] subsequently conducted another meta-analysis to evaluate the benefits and harms of higher versus lower F_I_O_2_ or target PaO_2_ in adult patients in ICU. They included a total of 10 RCTs with 1458 patients, and demonstrated that lower F_I_O_2_ or target PaO_2_ was associated with a marginal increase in the risk of mortality. In addition, higher F_I_O_2_ or target PaO_2_ was also associated with an increase in serious adverse events. However, the certainty in many of the included studies was very low, indicating that these results should be carefully interpreted.

By contrast, a subsequent RCT, the ICU-ROX study [58], investigated the benefit of conservative use of oxygen in adult patients who underwent mechanical ventilation. They enrolled a total of 1000 patients and demonstrated no significant benefit of conservative oxygen strategy (SpO_2_ 90–97%) compared with liberal oxygen strategy (SpO_2_ > 90%) for ventilator-free days or 180-day mortality. Another RCT, the LOCO2 study [59], investigated whether targeting lower PaO_2_ would improve outcome in patients with ARDS. They enrolled a total of 205 patients with ARDS, and demonstrated no benefit of conservative oxygen strategy (target PaO_2_, 55–70 mmHg; target SpO_2_, 88–90%) compared with liberal oxygen strategy (target PaO_2_, 90–105 mmHg; target SpO_2_, > 96%) on 28-day mortality. However, this study was prematurely stopped for safety concerns and a low likelihood of a significant difference.

Accordingly, the optimal strategy for supplemental oxygen is still controversial. In addition, these studies were predominantly conducted in the critically ill patient cohort. Therefore, the optimal strategy of supplemental oxygen for patients with ARDS remains unknown.

## ECMO

Although mechanical ventilation is the most common procedure to maintain oxygenation in patients with ARDS, the use of mechanical ventilation can potentially be associated with the occurrence of VILI, VIDD, or ventilator-associated pneumonia (VAP). ECMO, typically the venovenous (V-V) ECMO, would be the useful candidate for providing better oxygenation as well as minimizing the risk of VILI or VIDD. However, ECMO can also be associated with the potential risks including bleeding, thrombosis, and infection, an adequate selection of patients who can be favored from ECMO would be important.

### Patient selection

The generally accepted criteria for patient selection would be the guidelines published by the Extracorporeal Life Support Organization (ELSO) [60, 61]. ECMO should be considered for patients with the risk of mortality of > 50%, and is indicated for patients with the risk of mortality of > 80%. The risk of mortality of > 50% can be estimated by a PaO_2_/F_I_O_2_ ratio of < 150 on F_I_O_2_ > 0.9, and/or Murray score of 2–3. The risk of mortality of > 80% can be estimated by a PaO_2_/F_I_O_2_ ratio of < 100 on F_I_O_2_ > 0.9, and/or Murray score of 3–4. Intolerable hypercapnia and severe air leak syndromes would also be good indication for V-V ECMO. Contraindications include mechanical ventilation at high settings (i.e., F_I_O_2_ > 0.9, plateau pressure of > 30 cm H_2_O) for > 7 days, older age (with no specific cut-off value), and severe complications (i.e., irreversible organ damage).

Indications of V-V ECMO for patients with COVID-19 are generally consistent with the guidelines published by ELSO [62]. However, specific considerations for patient selection would be different, according to the presence or absence of pandemic situation. In a limited capacity, the resource-intensive support will be more preferential than the common recommendations [62]. Increased age seemed associated with increased mortality. Therefore, age of > 65, obesity (body mass index of > 40), duration of mechanical ventilation of > 10 days, Clinical Frailty Scale category of ≥3, and severe complications (i.e., chronic heart, liver, renal, neurological dysfunctions) would be relative or absolute contraindications.

### Initiation of ECMO

**Fig. 5:**
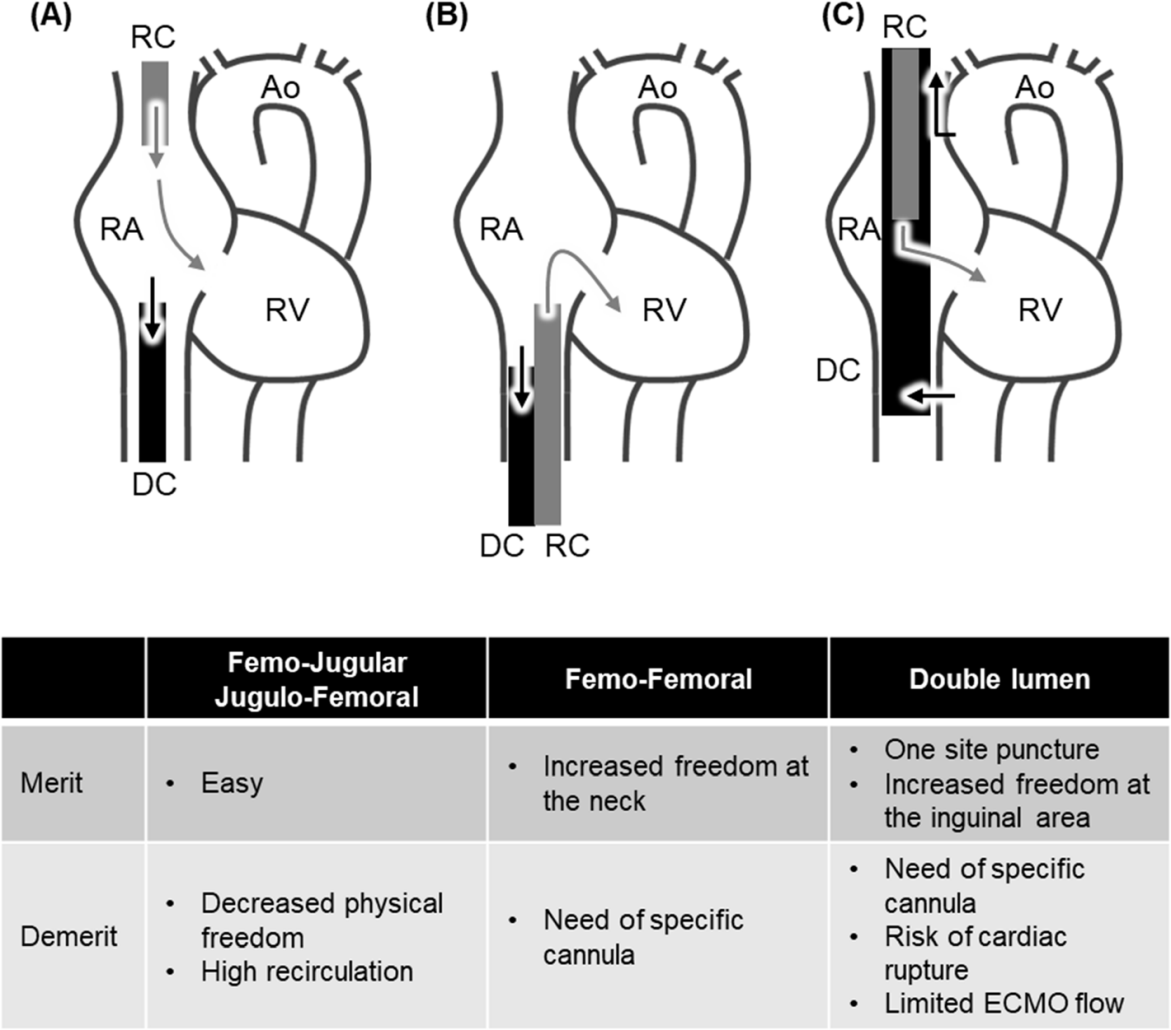
Various forms of V-V ECMO configurations and their characteristics. Various forms of V-V ECMO configurations can be used, according to their merits and demerits. **a** Femo-jugular approach: It is probably the most widely used configuration and has the advantage of being generalized and easy to use. However, since the neck and inguinal area are fixed, the physical liberality is low. In addition, recirculation is obvious. **b** Femo-femoral approach: Both two cannulas are placed in the vena cava inferior or the right atrium, approached from the left and right inguinal areas. Adequate diameter of vena cava inferior is essential. Side holes of return cannula must be centralized at the tip. **c** Double lumen cannula: It is usually approached through the right jugular vein, passing through the right atrium and locating the tip in the vena cava inferior. It has the advantage of complete freedom of both lower limbs. However, it is also associated with the disadvantages including the risk of malpuncture of the heart, inadequate positioning of the side hole of the return cannula not facing to the tricuspid valve, and the limited ECMO blood flow. RA, right atrium; RV, right ventricle; Ao, aorta; DC, drainage cannula; RC, return cannula

There are various forms of ECMO configuration (Fig. 5). One of the most important things to consider when starting ECMO is the cannula diameter. The use of a sufficiently large cannula minimizes blood cell destruction and limits the volume of blood transfusions. As the FACTT trial [63] has shown, the conservative fluid management strategy can improve lung function and shorten the duration of mechanical ventilation and ICU stay. The risk of inserting an additional drainage cannula at a later stage to further improve oxygenation should be minimized. Drainage cannulae are recommended to use a minimum of 23 Fr for adults. Return cannulae are recommended 19–23 Fr for adults.

The side holes of the cannula should be concentrated at the tip as much as possible. This is because the blood flow in the drainage cannula is larger at the proximal holes. This means that even if the tip of the drainage cannula is sufficiently located in the right atrium, the blood will be primarily drained from the superior or inferior vena cava through a drainage cannula with the side holes being widely distributed. Predominant drainage of blood from the superior or inferior vena cava can lead to inadequate blood flow.

In general, it is preferable to make no skin incision during cannulation. This is because even the smallest skin incision is at risk of inducing persistent bleeding and disrupting the systemic coagulation system due to the effects of heparin during ECMO management.

The material at the tip of the drainage cannula should contain metal wire, because V-V ECMO often requires high blood flow. The use of a drainage cannula made of soft material, which is often used in V-A ECMO, can cause the cannula to be collapsed and obstructed.

### Balance between oxygen supply and consumption

Various formulas are necessary to assess the balance between oxygen supply and consumption in organs during ECMO management. The oxygen content (CaO_2_), oxygen supply (DO_2_), and oxygen consumption (VO_2_) can be calculated by the following equations:

- CaO_2_ (mL/dL) = (1.34 × Hb × SaO_2_/100) + (0.003 × PaO_2_)
- DO_2_ (mL/min) = CaO_2_ (mL/dL) × cardiac output (L/min) × 10 (unit correction)
- VO_2_ (mL/min) = body surface area (m^2^) × 120

= 3 mL/kg/min (adults), 4–5 mL/kg/min (pediatrics), 6 mL/kg/min (neonates)

Normally, anaerobic metabolism does not occur and sufficient tissue oxygenation can be obtained if DO_2_ is more than three times that of VO_2_. These equations indicate that it is important to maintain not only SaO_2_, but also sufficient hemoglobin and cardiac output to supply sufficient oxygen to the peripheral tissues. The ECMO blood flow should be set so that the ECMO can supply all the required oxygen demand. Since oxygen demand can be calculated from the difference in oxygen supply between before and after the artificial lung, the required ECMO blood flow can be calculated using the following equation. If S_post_O_2_ is 100% and S_pre_O_2_ is 70%, the required ECMO flow rate can be calculated to be about 4.1 L/min.

- VO_2_ (mL/min) = D_post_O_2_ − D_pre_O_2_

= (1.34 × 12 × (S_post_O_2_ − S_pre_O_2_)) × ECMO blood flow × 10

- D_post_O_2_: Oxygen supply after the artificial lung
- D_pre_O_2_: Oxygen supply before the artificial lung
- S_post_O_2_: Oxygen saturation after the artificial lung
- S_pre_O_2_: Oxygen saturation before the artificial lung

- ECMO blood flow (approximation)

= 60–80 mL/kg/min (adults), 80–100 mL/kg/min (pediatrics), 120 mL/kg/min (neonates)

### Adjustment

F_I_O_2_ of V-V ECMO is always used at 1.0. A decrease in patient PaO_2_ is adjusted by increasing ECMO flow, and the target SpO_2_ is usually set to 85% or higher. The ratio of ECMO blood flow to sweep gas is approximately 1:1, and an increase in patient partial pressure of carbon dioxide (PaCO_2_) is adjusted by increasing the sweep gas flow. However, because rapid PaCO_2_ correction can induce cerebral vasoconstriction and cerebral hemorrhage, PaCO_2_ should be slowly adjusted.

The ventilator is usually set to the lung-protective ventilation setting (i.e., controlled ventilation mode, F_I_O_2_ of 0.21–0.40, plateau pressure of 20 cm H_2_O, PEEP of 10 cm H_2_O, respiratory rate of 5–10 breaths/min, respectively). The improvement of atelectasis by applying high PEEP is not important.

If SpO_2_ is decreased, it should be precisely evaluated whether it is caused by patient or ECMO factors. Patient factors include pneumothorax, increased oxygen consumption (e.g., sepsis), hemorrhage, decreased cardiac function, and pulmonary hypertension, while ECMO factors include poor performance of the artificial lungs and recirculation. Recirculation is a phenomenon in which oxygenated blood from the return cannula is drained from the drainage cannula without being used in the peripheral tissues. The recirculation rate can be calculated by the following formula (normal range, 0.3–0.5).

- Recirculation rate (*R*) = (S_pre_O_2_ − SvO_2_)/(S_post_O_2_ − SvO_2_)
  - SvO_2_: Oxygen saturation in central vein

Serial increase in S_pre_O_2_ or serial decrease in SaO_2_ is a sign of resuscitation. If the recirculation rate is high, the ECMO flow should be lowered or the tip distance between the drainage and the return cannulae should be increased.

### Complications

Major complications in the management of ECMO include bleeding, thrombosis, infection, and mechanical problems. Since hemorrhage can induce systemic coagulation failure, complete hemostasis is important if it is detected. Suturing and ablation, as well as blood transfusion, are necessary.

The prolonged ECMO management can be associated with the increased risk of infection. Pathogenic microorganisms include gram-positive cocci, gram-negative bacilli, and fungi. However, prophylactic administration of antibiotics is usually unnecessary, because no evidences supported that they reduce the risk of developing infection. Because several drugs, such as beta-lactams, carbapenems, and antifungals, can be adsorbed on the ECMO circuit, it is necessary to investigate the characteristics of each drug before administration.

Replacement of ECMO circuit is necessary when white or black thrombus becomes prominent in the artificial lung or cannula and the oxygenation capacity of the artificial lung starts to decline. Decreased platelets, elevated D-dimer, elevated thrombin-antithrombin III complex (TAT), and decreased fibrinogen may also be complementary indicators of deteriorated function of artificial lung. If the PaO_2_ in the return cannula is less than 300 mmHg, deteriorated function of artificial lung is strongly suspected. Repeated training is required to complete the replacement of the ECMO circuit in less than 1 min at the latest.

### Weaning from ECMO

Once the primary lung disease has sufficiently improved, withdrawal of ECMO will be attempted. First, change the ventilator settings to controlled ventilation mode, F_I_O_2_ of 0.4–0.6, plateau pressure of 20–25 cm H_2_O, PEEP of 10 cm H_2_O, and respiratory rate of 10–14 breaths/min, respectively. Next, the sweep gas flow rate is set to zero. After observing the patient for 30 to 120 min, the cannula will be removed after confirming no decrease in PaO_2_, increase in PaCO_2_, tachycardia, tachypnea, restlessness, or increase in respiratory workload. Normal skin sutures are sufficient for removal of cannulae.

### Clinical evidences

The EOLIA trial [64] an international prospective RCT that investigated the efficacy of V-V ECMO in patients with severe ARDS. A total of 249 patients were included, and 44 of 124 patients (35%) in the ECMO group and 57 of 125 (46%) in the control group had died at 60-day follow-up. The primary endpoint of 60-day mortality showed no significant benefit of early use of ECMO compared with a strategy of conventional mechanical ventilation (35% vs 46%; *p* = 0.09). However, this study was a crossover trial, which included a total of 35 patients (28%) in the control group received ECMO for refractory hypoxemia. If the crossover to ECMO in the control group was defined as treatment failure, the relative risk of treatment failure was 0.62 (95%CI, 0.47–0.82; *p* < 0.001).

Munshi et al. [65] conducted a meta-analysis to evaluate the benefit V-V ECMO in patients with ARDS. They included 5 studies, 2 RCTs, and 3 observational studies with a total of 773 patients. In the primary outcome of 60-day mortality, the use of V-V ECMO was associated with the better outcome compared with the use of conventional mechanical ventilation (34% vs 47%; RR, 0.73; 95%CI, 0.58–0.92; *p* = 0.008). Combes et al. [66] further collected individual patient data and conducted a meta-analysis of RCTs. Seventy-seven (36%) of the ECMO group and 103 (48%) of the control group had died on day 90. The risk ratio of 90-day treatment failure, defined as death for the ECMO group and death or crossover to ECMO for the control group was 0.65 (95%CI, 0.52–0.80). This meta-analysis of individual patient data also supported the previous meta-analysis by Munshi et al. [65].

Aoyama et al. [67] investigated the associations of currently available ventilatory strategies and adjunctive therapies with mortality to determine the best intervention for reducing mortality in adult patients with moderate to severe ARDS. They have included a total of 25 RCTs evaluating 9 interventions for Bayesian random-effects network meta-analyses. A total of 2686 of 7743 patients (35%) died within 28 days. They found that prone positioning was associated with lower 28-day mortality compared with lung-protective ventilation alone. In patients with severe ARDS, V-V ECMO was associated with lower 28-day mortality. This network meta-analysis did not find any associations of recruitment maneuvers or higher PEEP with mortality. Inhaled nitric oxide was associate with an increased risk for renal failure with no significant mortality benefit. The effect of neuromuscular blockade was similar to the results of the ROSE trial [68] and did not improve mortality in patients with moderate to severe ARDS.

## Conclusions

I have discussed the recent advances in the oxygen administration for patients with ARDS. Lung-protective ventilation remains the mainstay of respiratory management in ARDS, whereas the benefit of recruitment maneuver or high PEEP is not confirmed. Although ECMO has the potential to be a new procedure of oxygen administration for patients with ARDS, each physician should also become proficient in managing complications and troubles for improving patient outcome.

## Data Availability

Not applicable

## Abbreviations

ARDS: Acute respiratory distress syndrome
CaO_2_: Oxygen content
CI: Confidence interval
COVID-19: Coronavirus disease-19
CT: Computed tomography
DAD: Diffuse alveolar damage
DO_2_: Oxygen supply
D_post_O_2_: Oxygen supply after the artificial lung
D_pre_O_2_: Oxygen supply before the artificial lung
ECMO: Extracorporeal membrane oxygenation
ELSO: Extracorporeal Life Support Organization
F_I_O_2_: Fraction of inspiratory oxygen
HFNC: High-flow nasal cannula
HR: Hazard ratio
ICU: Intensive care unit
IQR: Interquartile range
NPPV: Non-invasive positive pressure ventilation
OR: Odds ratio
*P*_0.1_: Airway occlusion pressure
PaCO_2_: Partial pressure of carbon dioxide
PaO_2_: Partial pressure of arterial oxygen
PBW: Predicted body weight
PEEP: Positive end-expiratory pressure
*P*_occ_: Airway pressure during an end-expiratory airway occlusion
*P*_plat_: Plateau pressure
P-SILI: Patient self-inflicted lung injury
PSV: Pressure support ventilation
RCT: Randomized clinical trial
ROC: Receiver operating characteristics
SIMV: Synchronized intermittent mandatory ventilation
SpO_2_: Oxygen saturation
S_post_O_2_: Oxygen saturation after the artificial lung
S_pre_O_2_: Oxygen saturation before the artificial lung
SvO_2_: Oxygen saturation in central vein
TAT: Thrombin-antithrombin III complex
VAP: Ventilator-associated pneumonia
VIDD: Ventilator-induced diaphragm dysfunction
VILI: Ventilator-induced lung injury
VO_2_: Oxygen consumption
V-V: Venoveous
